# Urgent Psychiatric Consultations for Suicidal Ideation and Behaviors in Italian Adolescents during Different COVID-19 Pandemic Phases

**DOI:** 10.3390/jpm13071112

**Published:** 2023-07-09

**Authors:** Giulia Serra, Massimo Apicella, Maria Elena Iannoni, Monia Trasolini, Elisa Andracchio, Fabrizia Chieppa, Roberto Averna, Clotilde Guidetti, Gino Maglio, Antonino Reale, Stefano Vicari

**Affiliations:** 1 Child Neuropsychiatry Unit, Department of Neuroscience, Bambino Gesù Children’s Hospital, IRCCS, 00165 Rome, Italy; massimo.apicella@opbg.net (M.A.); mariaelena.iannoni@opbg.net (M.E.I.); monia.trasolini@opbg.net (M.T.); elisa.andracchio@opbg.net (E.A.); fabrizia.chieppa@opbg.net (F.C.); roberto.averna@opbg.net (R.A.); clotilde.guidetti@opbg.net (C.G.); gino.maglio@opbg.net (G.M.); stefano.vicari@opbg.net (S.V.); 2 Pediatric Emergency Department, Bambino Gesù Children’s Hospital, IRCCS, 00165 Rome, Italy; antonino.reale@opbg.net; 3 Department of Life Sciences and Public Health, Catholic University, 00168 Rome, Italy

**Keywords:** COVID-19, adolescent, mental health, suicide, attempt, depression, emergency care

## Abstract

Access to the emergency department (ED) for acute psychiatric problems, especially for suicide attempts (SA), has increased in the last decade. This increase has exceptionally accelerated after the COVID-19 pandemic. The aim of this project was to study the increase in acute psychiatric care demand of children and adolescents in the short and medium term after the pandemic, in relation to public health measures and in comparison with a pre pandemic reference period. We retrospectively studied 5445 child psychiatric (CP) consultations requested for any reason and for suicide attempt (SA), suicidal ideation (SI) and non-suicidal self-injury (NSSI) in a pediatric ED during three different pandemic periods in Italy (from March 2020 to May 2022) and compared them to a pre-pandemic reference period (from January 2018 to February 2020). Monthly CP consultations for any reason increased significantly by 2.2 times from 70.9 in 2018 to 157 in 2022 (*p* < 0.001). During the pandemic, monthly CP consultations for any reason increased significantly from 75/month in the first lockdown to 153/month in the second lockdown, remaining stable in the following year. CP consultations for SA increased significantly from 5/month in the first lockdown to 16/month in the second. Consultations for SI increased gradually but significantly from the pre-pandemic period to the end of the pandemic. Juveniles evaluated for SA during the pandemic vs. pre-pandemic more frequently attempted suicide by self-poisoning and less frequently by precipitation, and they were more likely to be diagnosed with a major depressive disorder. CP consultations for any reason and for suicide attempts significantly increased in the decade before the pandemic and peaked in the second lockdown period in Italy.

## 1. Introduction

Overall international suicide rates were recently estimated by the World Health Organization to be 10.6/100,000/year (per 100 k/year), accounting for 1.5% of all deaths. [1]. Suicide is the second leading cause of death among young persons aged 15–24 years worldwide, and suicide rates among adolescents in the international general population were estimated to be 3.77/100,000/year, with higher rates among older adolescents [2]. In Italy, the overall incidence rate for suicide has been estimated as 6.5/100,000 in 2015, a lower estimate in comparison to the European mean rates. For people aged under 24 years, the rate is of 1.4/100,000/year [3].

Over the past decade, annual suicide rates in the US increased by 57.4% among youths aged 10–24 [4]. According to a recent CDC report, there are now 10.7 suicides/100,000 persons in this age group, compared to 6.8/100,000 in 2007 [4,5]. Between 2010 and 2017, rates of suicide among adolescents aged 15–19 years increased in both sexes by 7.9%/year (95% CI: 4.80–11.2) in the UK [6]. Furthermore, while suicides among 5- to 11-year-olds are rare, they have also increased significantly between 2009 and 2018 [7]. Moreover, rates of suicide attempts among adolescents in the US increased by nearly 3-fold, and 40% more among girls than boys [8] during the last few decades. Rates of both suicide attempts and of non-suicidal self-harm increased by 22% between 2007 and 2016 among adolescents and young adults in Ireland, particularly among females and adolescents under 14 years of age, with an increasing lethality of methods employed [9]. Rates of these and other psychiatric problems, including depression, anxiety and substance abuse disorders among adolescents may be increasing the most in high-income countries [10]. 

Previous studies from our group [11,12] showed a highly significant 11-fold increase in the number of child psychiatric consultations ranging from 155 in 2011 to 1824 in 2021, corresponding, respectively, to 0.33% of total ED admissions in 2011 versus 4.15% of total annual ED admissions in 2021 [11,12]. Among those, the number of child psychiatry consultations for SA increased significantly by 33 times in the last decade, from 6 admissions for SA in 2011 to 200 admissions for SA in 2021, and the number of child psychiatry consultations for suicidal ideation increased by 32 times, from 4 admissions for SI in 2011 to 127 admissions for SI in 2021 (7.0% of total child psychiatry consultations) [11,12]. The number of psychiatric evaluations in ED increased significatively and stably during the study period from 2011 to 2021, whereas evaluations for SA increased significantly from 2011 to 2016 (APC from 2011 to 2016 of 64.1), were approximately stable from 2016 to 2020 (APC of 1.2 from 2016 to 2020) and then had a peak of 200 in 2021, during the coronavirus disease 2019 (COVID-19) pandemic (APC of 230.2 after 2020 in a two-joinpoints model). These time trends might reflect a secular trend in children and adolescents’ mental health with a superimposed effect of the pandemic, which might have had a significant impact on suicide attempts in the pediatric population. Among subjects admitted to our child psychiatry unit for suicide attempt, more than 80% were diagnosed with a major mood disorder [11]. 

A recent meta-analysis from our group [13] including 41 reports from 15 countries involving 104,801 juveniles (102,519 diagnosed with MDD, 2282 with BD), at risk for 0.80–12.5 years, estimated that the prevalence of juveniles with a major mood disorder who made at least one suicide attempt averaged 12.8% with a rate of 6.58%/year overall, equivalent to 6580/100,000 person exposure years [13]. In six studies, the estimated suicide rate was 125/100,000/year, to yield a ratio (A/S) of suicides/attempts of 52.6, indicating higher lethality than in the juvenile general population but possibly lower lethality than in adults diagnosed with major mood disorders [14]. 

We performed the present study: to investigate the potential effect of public health isolation measures and restrictions in different time phases of the pandemic between March 2020 and May 2022 in Italy on the number of child psychiatry consultation requested for self-harm, suicidal ideation and suicide attempts; and to evaluate the effect of the COVID-19 pandemic on the demographic and clinical characteristics of patients accessing the ED for suicide attempts.

## 2. Materials and Methods

### 2.1. Population and Setting

This is a retrospective chart review on patients admitted to the emergency department (ED) of Bambino Gesù Children’s Hospital (Ospedale Pediatrico Bambino Gesù IRCCS-OPBG) in Rome from 1 January 2018 to 31 May 2022. The method was described in a previous study [12]. 

OPBG is the largest pediatric hospital in Italy, with a second-level ED admitting patients coming not only from Rome and surrounding areas but also from other cities and regions. Access to the ED is free and open 24/24 h, 7/7 days. 

For the purposes of this study, we included data on patients (a) aged 0–18 years and (b) requiring a child psychiatry consultation in ED for any reason between 1 January 2018 to 31 May 2022. For subjects with repeated evaluations during the period analyzed, data from repeated admissions were included.

During the COVID-19 pandemic in 2020, the region where our hospital is located was locked down at the beginning of March 2020 with the rest of the country [15]. This is the reason why for our analysis we considered months form March 2020 to May 2022 as the period after the start of pandemic and the period from 2018 to February 2020 as the pre-pandemic reference period. For further analyses, we divided the period after the start of the pandemic in three time periods: Period 1, from March 2020 to November 2020, Period 2, from December 2020 to April 2021, and Period 3, from May 2021 to May 2022. We identified these intervals based on the dates of two changes in Italian government laws to contain the pandemic. Period 1 corresponded to the first lockdown period. In November 2020, Italian regions were divided by a new law in low, medium and high-risk zones based on public health risk, and the region where our institution operates was considered a low-risk zone, facilitating gradual resumption of different public activities [16], but many restrictions continued, so we referred to Period 2 as the second lockdown period. In April 2021, COVID-19 vaccines became available in Italy, and a new law allowed gradual resumption of many public activities [17], so we referred to Period 3 as the post-lockdown period.

### 2.2. Measures

Subjects entering the ED for a child psychiatric consultation are systematically assessed both clinically and by rating scales for the presence of suicidal ideation and behaviors. The Columbia suicide severity scale (C-SSRS) screening version is a screening instrument, consisting of a 6-item clinically administered interview, which is routinely administered to all subjects during the ED psychiatric consultation. 

The first two items explore the presence of suicidal ideation (passive thoughts or active ideation) during the last month and, if at least one is present, additional items are administered to further assess ideation and presence of suicidal behavior. The timing of the suicidal behavior is recorded. For the present study, suicidal behavior has been classified according to Posner [18]. An attempt is defined as any self-harming behavior resulting in any damage with non-zero intent to die, declared by the patient or evident from documented circumstances. Interrupted attempt is defined as a behavior inevitably leading to a suicide attempt, interrupted by a person or external circumstance before resulting in any damage. Self-interrupted/aborted attempt is defined as a behavior inevitably leading to a suicide attempt, interrupted by the individual autonomously before resulting in any damage. Preparatory behavior is defined as any act which is prepared for the imminent performance of a suicide attempt, including the access to a specific method and preparation to the perspective of own personal death. For the purposes of the present study, we considered a patient with an ED evaluation for suicide attempt (SA) as a patient with at least one attempt or interrupted attempt during the 7 days preceding the child psychiatric evaluation in ED. We considered a patient with an ED evaluation for SI as a patient with a wish to be dead/suicidal thoughts and method/some degree of intent/intent with specific plan in the last month before the access, as defined by the first 5 items of the CSSRS screening version, and without SA during the 7 days preceding the evaluation. We considered a patient with an ED evaluation for non-suicidal self-injury (NSSI) as a patient requiring psychiatric evaluation in ED for self-injurious behaviors occurred during the previous 7 days and performed deliberately without any intent to die [19].

For patients evaluated for SA, demographic data (sex, age), data on psychiatric diagnosis (DSM-5), previous admissions to the inpatient unit, number of previous SAs and method of the index SA episode were analyzed. ICD-10 codes were used to categorize SA methods (codes X60 to X84).

### 2.3. Statistical Analysis

Categoric variables have been presented as rate and number of observations. Continuous variables have been tested with Shapiro–Wilk test and, when not normally distributed, a non-parametric approach has been preferred. Continuous variables have been presented as mean and standard deviation (SD), when a parametric approach was applied, and as median, minimum and maximum values and interquartile range when a non-parametric approach was preferred. To compare two or more means, *t*-test for unpaired data or ANOVA were used, as appropriate. Post hoc Fisher LSD test was used for pairwise comparisons. To compare distributions, Kruskal–Wallis test has been used. To determine the statistical difference between incidence rates, the chi-square test was used for categorical variables. 

The analyses have been performed using Microsoft Excel and IMB SPSS v20.0.

## 3. Results

### 3.1. Child Psychiatry Consultations for Suicide Attempt and Suicidal Ideation from 2018 to 2022, Univariate Analysis

From 1 January 2018 to 31 May 2022, at the emergency department of the OPBG, 226,502 urgent medical evaluations were registered, requested for any reason by parents or caregivers of children and adolescents aged from 0 to 18 years old.

In the same period, 5445 Child Psychiatry (CP) consultations were performed at the emergency department of the OPBG, averaging 103 (SD 39.6) per month, requested for psychiatric reasons by parents or caregivers of children and adolescents aged 14.4 (3.12) years old on average and females in 59% of cases (Table 1). The average age of patients accessing the ED for CP consultations changed minimally but significantly over time (Table 1). 

The number of total CP consultations increased significantly by 2.2 times from 70.9 (SD 11.4) per month in 2018 to 157 (32.6) per month in 2022 (F = 51.2; *p* < 0.001; Table 1).

CP consultations requested for any reason concerning suicidal thoughts and behaviors and self-harm averaged 21.7 (SD 9.18) per month and increased significantly by 2 times from 15.8 (SD 5.36) per month in 2018 to 32.0 (SD 3.39) per month in 2022 (F = 19.0; *p* < 0.001; Table 1). 

Among those, CP consultations for suicide attempts averaged 8.36 (6.25) per month, peaking in 2021, with a mean of 16.7 (SD 7.18) per month, corresponding to almost four times the minimum mean number of suicide attempts per month registered in 2019 (4.33 (2.57); F = 20.7; *p* < 0.001; Table 1). CP consultations for suicidal ideation increased by 3 times from 5.67 (SD 2.10) per month in 2018 to 16.4 (SD 2.88) per month in 2022 (F = 13.9; *p* < 0.001; Table 1).

**Figure 1:**
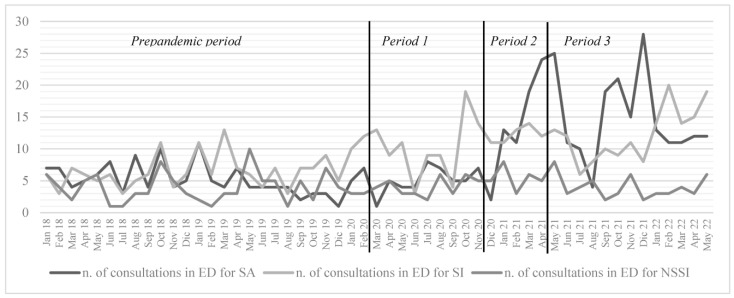
Number of consultations in the emergency department (ED) for suicidal attempts (SA), suicidal ideation (SI) and non-suicidal self-injury (NSSI) during the study periods.

Consultations for NSSI were 4.23 (SD 1.98) per month on average and did not change significantly over the period considered (Table 1). A line chart of the number of monthly consultations for SI, SA and NSSI during the study period is included in Figure 1.

### 3.2. Child Psychiatry Consultations for Suicide Attempt and Suicidal Ideation during Different Pandemic Periods

From 1 March 2020 to 31 May 2022 (pandemic period), 3366 Child Psychiatry (CP) consultations were performed at the emergency department of the OPBG. 

According to major differences in public health measures applied in Italy, we divided the post-pandemic period into three time intervals: Period 1, from March 2020 to November 2020; Period 2, from December 2020 to May 2021; and Period 3, from June 2021 to May 2022. 

**Figure 2:**
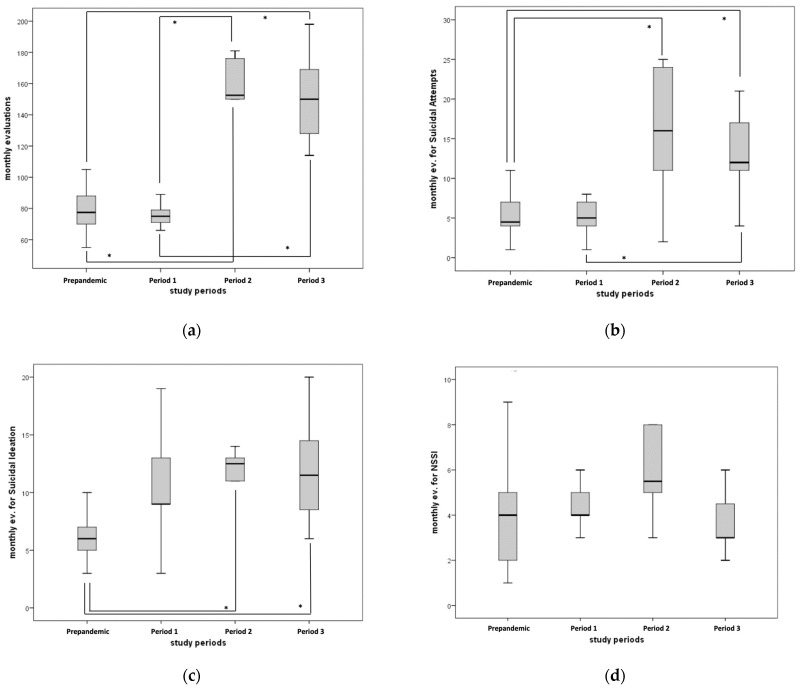
(**a**) Total psychiatric evaluations during pre-pandemic and different pandemic periods. Kruskal–Wallis for comparison of the four groups (Stat. 31.4; *p* < 0.001). Pairwise comparisons: Period 1 vs. Period 2 (Stat. −25.83; *p* = 0.009); Period 1 vs. Period 3 (Stat. −26.88, *p* < 0.001); pre-pandemic vs. Period 2 (Stat. −23.81; *p* = 0.004); pre-pandemic vs. Period 3 (Stat. −24.86, *p* < 0.001). (**b**) Psychiatric evaluations for suicide attempt during pre-pandemic and different pandemic periods. Kruskal–Wallis for comparison of the four groups (Stat. 23.35, *p* < 0.001). Pairwise comparisons: Period 1 vs. Period 3 (Stat. −21.04 *p* = 0.011); pre-pandemic vs. Period 2 (Stat. −20.67, *p* = 0.017); pre-pandemic vs. Period 3 (Stat. −22.32, *p* < 0.001). (**c**) Psychiatric evaluations for suicidal ideation during pre-pandemic and different pandemic periods. Kruskal–Wallis for comparison of the four groups (Stat. 19.67, *p* < 0.001). Pairwise comparisons: pre-pandemic vs. Period 2 (Stat. 22.96, *p* = 0.006); pre-pandemic vs. Period 3 (Stat. −19.37, *p* = 0.002). (**d**) Psychiatric evaluations for non-suicidal self-injury during pre-pandemic and different pandemic periods. Distributions differences not significative, Kruskal–Wallis: 5.32, *p* = 0.150. * = *p* < 0.05 for the comparison.

The number of total CP consultations increased significantly by two times from 75/month (interquartile range 68.5 to 84.0) in Period 1 to 153/month (interquartile range 132 to 177) in Period 2, and then they remained stable at 150/month (interquartile range 124 to 174) in Period 3 (Kruskal–Wallis: 31.4, *p* < 0.001) (Table 2, Figure 2a).

The number of CP consultations for suicide attempts increased significantly by three times from 5/month (interquartile range 4.00 to 7.00) in Period 1 to 16/month (interquartile range 8.75 to 24.5) in Period 2, and then they remained stable at 12 evaluations/month (interquartile range 11.0 to 18.0) in Period 3 (Kruskal–Wallis 23.4; *p* = 0.003) (Table 2, Figure 2b).

The number of CP consultations for suicidal ideation increased significantly and constantly from 6/month (interquartile range 5.00 to 7.50) in the pre-pandemic period, to 9/month (interquartile range 6.50 to 13.5) in Period 1, 12.5/month (interquartile range 11.5 to 13.3) in Period 2 and 11.5/month (interquartile range 8.25 to 14.8) in Period 3 (Kruskal–Wallis 19.7; *p* < 0.001) (Table 2, Figure 2c). In particular, the median monthly consultations for suicidal ideation significantly increased by two times from the pre-pandemic period to Period 2 and Period 3 (Table 2; Figure 2c). 

No significant differences were found in the median number of evaluations for NSSI (Kruskal–Wallis 5.32, *p* = 0.150; Table 2, Figure 2d). 

### 3.3. Access to the ED for Suicidal Attempts from 2018 to 2021

Between January 2018 and December 2021, we registered 384 admissions to the emergency department requiring a CP consultation for suicide attempt or 8.36 (SD 6.25) per month. 

Of those, 323 (84.1%) admissions were made by females, and the average age of juveniles accessing at the ED was 15.8 (SD 1.48) years. Forty-eight percent of adolescents who accessed the ED had a history of at least one previous urgent evaluation at the OPBG ED for any reason, including an average of 1.41 (SD 3.81) previous urgent psychiatric evaluations (Table 3). 

The most common method for attempting suicide was self-poisoning in 249 cases (64.8% of total ED admissions for SA), followed by precipitation from a height in 16.4%, attempt at suffocation in 9.11%, deep cutting in 6.77%, and vehicle accident in 2.08% of cases (Table 3). 

Among subjects presenting to the ED for an urgent CP consultation for attempting suicide, 295 (76.8% of total ED admissions for SA) were discharged from the ED with a diagnosis of mood disorder, including a major depressive episode in 214 (55.7% of total ED admissions for SA), bipolar disorder in 15 (3.91%) and mood disorder NOS in 66 subjects (17.2% of total ED admissions for SA). Overall, 26 (6.77%) subjects had a primary diagnosis of post-traumatic stress disorder, 23 (5.99%) a diagnosis of oppositional defiant disorder, 16 (4.17%) a diagnosis of psychosis, 9 (2.34%) a diagnosis of eating disorder, and 6 (1.56%) a diagnosis of anxiety disorder. Thirty-one subjects (31%) had at least one documented comorbid psychiatric diagnosis (Table 3). 

### 3.4. Suicide Attempts Pre- versus Post-COVID-19 Pandemic, Bivariate Analysis

We analyzed the number of ED admissions for SA, as well as methods of attempts and admission diagnosis stratifying the sample depending on the period of admission at the ED during the pre-pandemic (from 1 January 2018 to 8 March 2020) versus the pandemic period (from 8 March 2020 to 31 December 2021). 

The number of CP consultations for suicide attempts per month increased significantly by two times from 5.23 (SD 2.42)/month before lockdown to 11.37 (SD 7.29)/month thereafter (mean difference 6.14, CI 9.16–3.12; t = 4.14, *p* < 0.001)

Self-poisoning (ICD-10 X60 to X69) rate increased from 57.4% pre-pandemic to 68.9% post-pandemic (χ^2^ = 5.18, *p* = 0.023), and precipitation (ICD-10 X80) decreased from 22.8% pre-pandemic to 12.9% (χ^2^ = 6.27, *p* = 0.012) in the pre-pandemic period versus post-pandemic. 

Among subjects assessed for SA, the diagnosis of any mood disorder increased from 64.4% pre-pandemic to 83.1% post-pandemic (χ^2^ = 15.3, *p* < 0.001), including a significant increase in the diagnosis of major depressive episode from 41.2% pre-pandemic to 63.7% post-pandemic (χ^2^ = 18.1, *p* < 0.001, Table 2). Conversely, the diagnosis of oppositional defiant/conduct disorder decreased (10.3% vs. 3.63%; *p* = 0.008) in the pre-pandemic versus post-pandemic period. Mixed manic episodes (3.68% vs. 4.03%; *p* = 0.86) and depressive disorder not otherwise specified (20.6% vs. 15.3%; *p* = 0.19) were not significantly different. Further details on this population are provided in Table 3.

## 4. Discussion

We already reported data on a significant secular trend towards the increase in child psychiatry (CP) consultations observed in Italy at the emergency department of the Bambino Gesù Children’s Hospital in the last decade [12]. Specifically, child psychiatry consultations for suicide attempts increased significantly by 33 times from 2011 to 2021, with a first peak in the increase between 2011 and 2016 and a second one in 2021 during the COVID-19 pandemic [12]. The present study focuses on the second peak in child psychiatry consultations observed during the pandemic period and persisting after the gradual resumption of community activities in Italy after the first period of lockdown. 

The 27-month pandemic period considered in this study includes 15 months of more severe restrictions (Period 1 and Period 2) and the following 12 months characterized by the gradual reopening of activities (Period 3), compared to a matched reference period consisting of the preceding 26 months (pre-pandemic period, from January 2018 to February 2020). 

Monthly CP consultations for any reason increased by more than two times from 2018 to 2022 (70.9 versus 157; *p* < 0.001, Table 1), with stability between the pre-pandemic period versus Period 1, and a significant two-fold increase between Period 1 and Periods 2 and 3 (70.5 versus 150–153 CP/month, respectively, *p* = 0.009 and *p* < 0.001). The same trend was observed for CP consultations for suicide attempts, which remained stable between pre-pandemic and Period 1 (4.50 versus 5.00 CP/month, Stat. −1.282; *p* = 0.829) and peaked significantly by almost four times during Period 2, remaining stable during Period 3 (Table 2, Figure 2b). Consultations for suicidal ideation increased significantly and gradually from pre-pandemic to Period 2 and 3 (Table 2, Figure 2c). 

Other authors reported data about the stability of the number of CP consultations during the first pandemic lockdown, and the same trend was observed and reported after other ecological disasters [20,21]. During the pandemic, we experienced a unique circumstance, and juveniles were particularly affected. Social interactions abruptly changed quantitatively and qualitatively with an obliged preference for virtual relationships [22]. Abrupt school closing probably played an important role in stress perception among youngers, with adverse effects on children health and well-being already in the short term [23]. Economic uncertainty among families with a lower socio-economic status, alteration of sleep habits with inversion of the circadian rhythms, decreased physical activity, unhealthy eating habits, and possible difficulties in family relationships including exposure to intrafamilial violence/traumas are all possible reasons for the adverse health outcomes relating to the pandemic lockdown [24]. Several other studies reported an increase in ED psychiatric consultations from suicide attempts after the spring season of 2020, and adolescents have been reported as the most vulnerable group [21,25,26,27,28]. The present study showing an increase in CP consultations for suicidal ideation and attempts confirms the results of a previous Italian study, providing information on the persistence of this increase during the year after gradual resumption of normal activities for both adults and juveniles. 

The “tardive” increase in CP consultations for suicide attempts calls for a comment. The Period 2 (or second lockdown period) was characterized in Italy by the gradual resumption of some work activities for adults but permanent restrictions of school reopening, with a high percentage of online classes. During Period 3, school reopening along with the resumption of performance requests and among-peers confrontation and competition might have favored an increase in suicide attempts in youth [28]. 

While a recent Italian study [28] found an increase in NSSI during the second lockdown, our data failed to detect any significant variations in ED presentations for this indication. This is probably due to a limitation in our methodology. We categorized ED admissions for the main reason of admissions and did not systematically analyze NSSI as a bystander comorbidity in patients presenting mainly for SI or for SA. However, as ED consultations for minor urgency for any medical reason decreased during the pandemic, our data may also result from a real relative decrease in NSSI admissions. Of note, NSSI is considered as a less severe and chronic psychiatric symptom compared to suicidal ideation and attempt and a minor urgency for ED presentation [29]. 

In the last part of this study, we focused on the SA characteristics, in particular methods of attempts and psychiatric diagnosis, to study the differences between attempters after the lockdown period in comparison to the previous pre-pandemic reference period. As regards to methods, we found self-poisoning to have increased and represent more than two-thirds of post-pandemic attempts, while precipitation almost halved. This trend of an increased prevalence of self-poisoning during the COVID-19 pandemic is in line with other studies performed in clinical settings similar to ours [30]. Self-poisoning has been reported to be linked to non-suicidal self-injury and to relate to an attempt at self-medication and/or a dysfunctional self-regulation attitude [31]. Self-poisoning in adolescents is usually less lethal than in adults, and is considered to related to a lower suicidal intent compared to other suicidal methods [32]. The high prevalence of switching between NSSI and self-poisoning in repeated ED admissions [33] and a continuum of psychopathological features between self-poisoning and NSSI has been described in pre-pandemic cohorts [34]. The surplus suicide attempts by self-poisoning after the pandemic, not matched by a parallel increase in NSSI, may represent a different subset of attempts. There might be also more ambivalence in the suicidal intent of juveniles attempting suicide by self-poisoning, and setting the criterion for suicide attempt as a self-harm behavior with a “non-zero intent” to die might have limited a distinction between suicide attempts and non-suicidal self-harm performed by self-poisoning [35]. 

Among juveniles assessed for suicide attempt post-pandemic, the diagnoses of major depressive disorders increased, representing the most frequent diagnosis among attempters, while oppositional defiant/conduct disorder decreased. Bipolar disorder diagnoses did not change. This increase further supports a true worsening in the severity of depressive symptoms in youths accessing psychiatric care. 

Comorbid neurodevelopmental diagnosis (such as ADHD, intellectual disability) decreased among attempters. ADHD and intellectual disability were also reduced among second lockdown patients from a recent Italian study [28] where the authors discuss that reduced help-seeking and decreased hospital admissions of disabled patients suggest that “in time of crisis, people with intellectual disability may have been the least supported patients”.

Our study has the strength of presenting data from an ED with a large volume of pediatric patient admissions covering a broad period. Our institution is a referral center in its region and admits patients from surrounding Italian regions, where there are fewer or no pediatric psychiatric inpatient units, so differences described between pre- and post-pandemic periods concern a large sample of the Italian juvenile population.

Our results should be interpreted considering the following limitations: the lack of control for factors reported to have an impact on post-COVID-19 mental health such as socio-economic status, reduction in physical activity and fear of contagion; retrospective data collection; and the absence of detailed psychopathological evaluations of subjects evaluated for suicide attempts. Furthermore, as our institution is a referral center for its own territorial district and for the surroundings, this setting may be biased towards overestimation of the proportion of the most severe cases among all CP consultations, and generalizability may be limited.

## Figures and Tables

**Table 1:** Number of urgent evaluations per month at the emergency department of OPBG from 2018 to 2022. Monthly psychiatry evaluations are presented as mean (standard deviation). Comparison of the mean monthly psychiatric evaluations in ED, reason for admissions and demographics during study period (January 2018 to May 2022). Univariate ANOVA has been used to compare means.

	2018	2019	2020	2021	2022	Total	*p*-Value (F)
Total psychiatric evaluations	70.9 (11.4)	88.3 (17.6)	77.3 (11.8)	152 (21.1) *	157 (32.6) *	103 (39.6)	<0.001 (51.2)
Evaluations for suicidal ideation, attempts and self harm	15.8 (5.36)	15.6 (4.70)	19.5 (5.23)	31.8 (8.49) *	32.0 (3.39) *	21.7 (9.18)	<0.001 (19.0)
Suicide attempts	6.00 (2.22)	4.33 (2.57)	5.00 (2.09)	16.7 (7.18) ^$^	11.8 (0.84) *	8.36 (6.25)	<0.001 (20.7)
Suicidal ideation	5.67 (2.10)	7.08 (2.81)	10.3 (4.25) **	10.6 (2.43) **	16.4 (2.88) ^$^	9.17 (4.24)	<0.001 (13.9)
Non-suicidal self-injury	4.17 (2.29)	4.17 (2.52)	4.17 (1.27)	4.58 (2.11)	3.80 (1.30)	4.23 (1.98)	0.96 (0.15)
Age	14.2 (3.54)	14.0 (3.49)	14.5 (3.10) **	14.6 (2.81) **	14.7 (2.63) **	14.4 (3.12)	<0.001 (8.19)

* Post hoc LSD test with *p* < 0.05 for comparison with 2018, 2019 and 2020. ** Post hoc LSD test with *p* < 0.05 for comparison with 2018 and 2019. ^$^ Post hoc LSD test with *p* < 0.05 for any one-by-one comparison with another year. ED = emergency department; SA = suicidal attempt; SI = suicidal ideation; NSSI = non-suicidal self-injury.

**Table 2:** Monthly psychiatric evaluations in ED versus pandemic periods. Monthly psychiatric evaluations in ED requested for any psychiatric reason, for suicide attempt, suicidal ideation and non-suicidal self-injury from January 2018 to May 2022, divided by pandemic period. Data are presented as median (interquartile range). Kruskal–Wallis test has been used to compare the four groups.

	Pre-Pandemic	Period 1	Period 2	Period 3	Total	Kruskal–Wallis; *p*-Value
Total	77.5 (69.8–88.0)	75.0 (68.5–84.0)	153 (132–177)	150 (124–174)	86.0 (73.5–138)	31.4; *p* < 0.001
Suicide attempts	4.50 (4.00–7.00)	5.00 (4.00–7.00)	16.0 (8.75–24.3)	12.0 (11.0–18.0)	7.00 (4.00–11.0)	23.4; *p* < 0.001
Suicidal ideation	6.00 (5.00–7.50)	9.00 (6.50–13.5)	12.5 (11.0–13.3)	11.50 (8.25–14.8)	9.00 (6.00–12.0)	19.7; *p* < 0.001
Non-suicidal self-injury	4.00 (2.00–5.00)	4.00 (3.50–5.50)	5.50 (4.50–8.00)	3.00 (3.004.75)	4.00 (3.00–5.00)	5.32; *p* = 0.150

**Table 3:** Analyses on 384 patients evaluated for suicide attempt in ED between January 2018 and December 2021. Continuous variables are presented as mean (standard deviation) and categorical variables as percentage (number). Comparisons are made with *t*-test for continuous variable and chi-square for categorical variables.

	Pre-Pandemic N = 136	During Pandemic N = 248	Total N = 384	Statistic	*p*-Value
Age	16 (1.58)	15.7 (1.42)	15.8 (1.48)	1.796	0.073
Female sex	85.3% (116)	83.5% (207)	84.1% (323)	0.219	0.640
Anamnesis of at least one previous psychiatric evaluation in ED for any reason	50.0% (68)	46.8% (116)	47.9% (184)	0.366	0.545
Number of previous psychiatric evaluation in ED for any reason	1.81 (4.65)	1.19 (3.23)	1.41 (3.81)	1.379	0.169
Methods of SA					
Cutting, ICD-10 X78	6.62% (9)	6.85% (17)	6.77% (26)	0.008	0.929
Precipitation, ICD-10 X80	22.8% (31)	12.9% (32)	16.4% (63)	6.265	0.012
Self-poisoning, ICD-10 X60-69	57.4% (78)	68.9% (171)	64.8% (249)	5.183	0.023
Suffocation, ICD-10 X70-71	10.3% (14)	8.47% (21)	9.11% (35)	0.354	0.552
Vehicle accident, ICD-10 X81	2.94% (4)	1.61% (4)	2.08% (8)	0.760	0.383
Psychiatric diagnosis at admission					
Any mood disorder diagnosis	65.4% (89)	83.1% (206)	76.8% (295)	15.321	<0.001
Bipolar and related disorders	3.68% (5)	4.03% (10)	3.91% (15)	0.030	0.863
Major depressive episode	41.2% (56)	63.7% (158)	55.7% (214)	18.076	<0.001
Mood disorder not otherwise specified	20.6% (28)	15.3% (38)	17.2% (66)	1.711	0.191
Psychosis not otherwise specified	6.62% (9)	2.82% (7)	4.17% (16)	3.168	0.075
Post-traumatic stress disorder	10.3% (14)	4.84% (12)	6.77% (26)	4.141	0.055
Anxiety disorder	0.735% (1)	2.02% (5)	1.56% (6)	0.937	0.333
Oppositional defiant disorder/conduct disorder	10.3% (14)	3.63% (9)	5.99% (23)	6.929	0.008
Eating disorder	3.68% (5)	1.61% (4)	2.34% (9)	1.634	0.289
Presence of at least one comorbid neurodevelopmental disorder	14.7% (20)	4.44% (11)	8.07% (31)	12.484	0.001

## Data Availability

The data presented in this study are available on request from the corresponding author. The data are not publicly available.
